# The magnitude of parental physical harms: a descriptive report of 76 abusive children in Isfahan

**Published:** 2024-07

**Authors:** Gholamali Dorooshi, Babak Bakhshaie, Behgat Keshavarzi Moghadam, Ali Soleimanpour, Ehsan Eslaminia, Sarah Keshavarzi Moghadam, Ahmad Bagheri Moghaddam, Zahra Jahanbin, Shiva Samsamshariat

**Affiliations:** ^*a*^ Clinical Toxicology Department, School of Medicine, Isfahan Clinical Toxicology Research Center, Isfahan University of Medical Sciences, Isfahan, Iran.; ^*b*^ Legal Medicine Center, Legal Medicine Organization, Isfahan, Iran.

**Keywords:** Physical Harm, Parental Victimization, Forensic Medicine

## Abstract

**Background:**

Victimization of parents by their children is a widespread phenomenon. However, there is a shortage of literature regarding the abusive behavior of children in Iranian society. The primary focus of this study was to highlight the magnitude and nature of the abusive behavior of a handful of Iranian children toward their parents.

**Methods:**

The sample study comprised 76 cases referred to the Isfahan Forensic Department (IFD) from September 2015 to October 2016. Data were computer analyzed using SPSS-21 by running a couple of descriptive-analytical tests.

**Results:**

Most of the victims were elderly mothers above 60 years old. The victims were mostly injured in the neck and head. The majority of the offenders were between 20 to 30 years old, unemployed, with a family history of substance abuse. Significant differences (p less than 0.05) were observed in some variables: gender (women), age (over 60 years), harassment in the residence, having financial authority, 4 children or more (in victims group) and unemployment, single, age between 21-30 years (in offenders group).

**Conclusions:**

Children’s abusive behavior towards their parents is a multifactorial phenomenon. Family constitution and background appeared to play a pivotal role in shaping the offenders’ social behavior and reaction to their parents. Nonetheless, there is a need to explore further the possible causes of parental victimization.

## Introduction

Violence against the elderly by family members or parent abuse is deeply rooted in child reading practices and child abuse. A growing body of research indicates that individuals who are raised in violent family environments may exhibit a higher propensity for engaging in retaliatory behaviors and demonstrating hostility towards their parents during adolescence.^1^ These children can victimize their parents physically, mentally, socially, and financially.^2^ They tend to insult, humiliate blackmail, impose financial debt, and create situations that are embarrassing for parents, which can result in their social isolation.^3^ Psychological and social impacts of such behaviors on parents are enormous some of which include hopelessness, self-blame, lack of self-esteem and a lack of attitude, feelings of despair, frustration, social phobia, general anxiety, guilt, feeling humiliated, sense of dissatisfaction and uselessness.^4^ The elderly parent is fragile and such horrifying experiences can disturb their state of homeostasis and physical function.^5^ There is no consensus regarding children’s behavior. Some studies have found fault with families and others blamed communities for such atrocities.^6^

The World Health Organization estimates that 4-6% of the elderly worldwide experience a form of misconduct.^7^ A Study in Iran has reported that one of the reasons for not satisfying the mental needs of the elderly was the misconduct by family members.^8^ In a study conducted by Heravi-Karimooi in Tehran on Health-related quality of life among abused and non-abused elderly people, the most damage to parents was emotional and psychological, and physical trauma was less common.^9^ Recently, cases of disrespect and mental harassment, and especially the physical harm of parents by children have been rising and many of them are not just elderly parents, but also middle-aged parents and grandparents. Parent abuse in Iran has not been investigated for various reasons, including cultural issues, and so far, there are no studies regarding the extent and characteristics of the harassed parents. Therefore, in this study, we aimed to shed light on the frequency of physical harm caused by physical abuse of parents by children.

## Methods 

In this cross-sectional study, following the approval of the research ethics code (No.: P.1393.14481) granted by the Research Ethics Committee of the Isfahan Forensic Medicine Organization, we identified 76 eligible cases of ‘parental victimization’ referred to the Isfahan Forensic Department (IFD) in Isfahan, Iran, during the period from September 2015 to October 2016. At the commencement of the study, we conducted registrations and documented the victim's life history through interviews with the victim and informed individuals. The inclusion of cases was contingent upon certification from a forensic physician. In instances where ambiguity or uncertainty concerning diagnosis arose, the case was excluded from the sampling list. All eligible cases were cataloged, and during the explanation of the study objectives, participants were assured that their information would be treated with the utmost confidentiality. To prevent any future complications, we obtained written consent from the relevant parties. An interview schedule was utilized, incorporating both closed and open-ended questions that gathered demographic data regarding the victim and the offender, as well as details about the nature of the offense, its location, and the time of occurrence. The data were analyzed using SPSS version 21, employing several descriptive statistical tests. The Chi-Square Test was implemented to compare the frequency of each variable across the studied groups. This study was approved by the moral committee of the IFD.

## Results

The victims were typically elderly married women who were living with their children. Their life history indicated that the victims did not necessarily live with their spouses (Table 1). The offenders were typically single young male children, with secondary education, and lacking a source of income. As previously noted, these offenders were often exposed to turbulent familial environments characterized by parental physical and emotional detachment. Surprisingly, a significant proportion of the offenders were grandchildren (Table 2 ).

**Table 1:** Demographic characteristics of the victims (N=76)

Variable	Frequency	Percentage
**Gender**		
Male	30	39.5
Female	46	60.5
**Age Group(Year)**		
20-40	4	5.0
41-50	12	15.0
51-60	20	25.0
≥	40	55.0
**Marital Status**		
Married	40	52.6
Divorced	12	15.9
Widow	24	31.5

**Table 2:** Table-2: Demographic characteristics of the offenders (N=76)

Variable	Frequency	Percentage
**Gender**		
Male	60	79.0
Female	16	21.0
**Age Group (Year)**		
10-20	7	10.0
21-30	44	58.0
31-40	16	21.0
≥41	9	11.0
**Marital Status**		
Married	22	27.0
Divorced	8	11.0
Single	46	62.0
**Educational Status**		
Primary	10	14.0
Secondary	44	57.0
≥Graduation	22	29.0
**Occupational Status**		
Employed	28	37.0
Unemployed	48	63.0

In terms of physical injury, all the harassed parents were beaten up. Forty-six percent of the parents had bruises, 12% had scratches and rubbing, 7% had tears, 11% had swelling, 4% had fractures, and 25% had more than one type of beating on their body. In 77% of cases, parents were beaten more than once, and none of the cases led to admission to the hospital. Fifty- eight percent of the cases had not had a forensic medical examination, and most of them feared going to forensic medicine offices. Most of the parents were beaten in the head and neck (32.6%), and in 87% of the cases, more than one area was injured. Thirty-one percent of the parents once a day, 6% of the parents once a week, 11% of the parents once a month, and 51% of the parents regularly met with their children. In terms of the four aspects of care, food, grooming and health care, drug delivery, and entertainment, the parents were responsible for these in 78%, 90%, 82% and 75% of the cases, respectively. All the parents were harassed by their childern under mental and psychological pressure and neglect. All the parents were mistreated by the child and 57% of the parents were driven from their homes or family members.

About 15.7% of the parents were public employees, 57.3% of the parents were pensioners, and 16% of the parents had private businesses. Sixty-three percent of the parents forced by their children to pay, 21% were forced to change wills and 27% were forced to delegate to their children. Furthermore, in terms of the economic aspects, 26% of the parents did not have financial authority, and 74% of the parents were financially abused by their children. Significant differences (p<0.05) were observed in some variables: gender (women), age (over 60 years), harassment in the residence, having financial authority, 4 children or more (in victims group) and unemployment, single, age between 21-30 years (in Offenders group). No significant differences were observed in Marital Status in victims as well as the presence or absence of addiction in offenders.

## Discussion

Annoying behaviors such as complaints or ongoing protests from family members to each other and the behavior of one of them can affect other members. An individual who dedicates considerable energy to the support and care of others, in the absence of acknowledgment for their efforts, is unlikely to experience a sense of restfulness. On the other hand, many of the issues that naturally control violence against strangers are not in the family environment. People are not punished for their brutal behaviors against family members, and social attitudes are the secret nature of family violence and the fundamental injustice in family communication creates the atmosphere in which violence and cruelty are accepted.^10,11^ Research has shown that dynamics within the family represent a distinctly personal and private relationship. Among various domestic behaviors, it is noteworthy that violent and sexual behaviors are regarded as the most privileged forms of behaviors. In other words, the prevailing attitude of most societies is that the family is a private environment, and others are not allowed to interfere in the personal affairs of individuals. All of the above issues confirm that violence and abuse in the family are broadly common in the community.^12^ Numerous theories have been proposed to elucidate the factors contributing to violence against parents and the elderly. One such theory is the situational theory, which highlights various caregiver-related issues, including inadequate care, substance addiction, loss of employment, and other economic difficulties that may precipitate the mistreatment of the elderly population. Fatigue and high stress levels of the caregiver also create a favorable environment for abuse.^13^ Adherence to ethical values by the caregiver of elderly parents can have a more decisive and deterrent effect than part of the legal approach to preventing parental mismanagement. Awareness of the family and replacement of the respect and dignity of the elderly can play an important role in families with the violence and neglect that inevitably occurs in the industrial community. Numerous longitudinal epidemiological studies provided valuable insights into the causes of the declining importance and value of parents and the elderly in modern societies .These insights include reducing belief in the traditions and competence of the elderly, changing the mechanisms of knowledge and experience transmission, reducing the participation of the elderly in the economy, separation from the family nucleus by children, reducing the function of new families, increasing the gap between generations and changes in the architecture and living space of houses.^14^ In a similar study by Fawzi et al. in Egypt, 150 outpatients who were known to be psychotic were investigated in terms of the frequency of parental abuse among them with questionnaires and interviews.^15^ In that study, 40.7% of the cases hurt their parents, and similar to our findings, most of the injured parents were mothers. The risk factors associated with parental involvement in this study were the female gender of the parent, male genital mutilation, and childhood trauma, which is similar to the results of our study. However, there are significant differences between our findings and their study, which may in part be clarified by differences in the inclusion criteria of their study because they only studied patients with psychosis. In another study by Ibab et al.^16^ 103 individuals referring to a juvenile delinquency court in Spain were investigated. The results of their study showed that 33% of the participants in the study committed violence against their parents. Although similar to our study, the highest rate of violence was against mothers (95%), there is a significant difference between the rate found in our study (60.5%) and that reported by them. Similarly, most of the parent abusers were boys. Contrary to our study, the most prevalent age range was reported to be between 14 to 16 years of age, while in our study most of the subjects were between 20 and 30 years of age, with an age range of 10-20 years accounting for only 10% of the cases. This difference may have explained by the type of participants in the study, as well as differences in the culture of different countries. The higher numbers of retired parents are probably due to the better socio-economic situation and the possibility of their further referral. The prevalence of secondary level or higher among offenders can be attributed to the educational attainment within the urban population of Iran. To the best our knowledge, this is the first study conducted in Iran that investigates the frequency and profile of physical injuries reported in parental abuse cases referring to forensic medicine.

## Conclusion

The results of the study showed that, in addition to older parents, their children might also harm younger parents. Most of the harassers were single, unemployed, and addicted. Therefore, to prevent the breakdown of the family foundation and the moral principles of the community, necessary actions for the elimination of youth unemployment and addiction issues are essential. However, further studies will be required to determine the risk factors of parent abuse in various cultures.

**Acknowledgements**

We extend our gratitude to the personnel of the Isfahan Legal Medicine Center for their support and encouragement throughout this endeavor.

**Author contributions**

 Patient care, data acquisition, literature review, drafting, and submitting the manuscript were conducted by all authors. Gholmali Dorooshi critically reviewed the draft for significant intellectual content. Furthermore, Gholmali Dorooshi revised the manuscript for English style and language. All authors reviewed and approved the final version prior to submission.
